# Comparison of Mitochondrial Genome Expression Differences among Four Skink Species Distributed at Different Latitudes under Low-Temperature Stress

**DOI:** 10.3390/ijms251910637

**Published:** 2024-10-02

**Authors:** Lemei Zhan, Jingyi He, Lingyi Ding, Kenneth B. Storey, Jiayong Zhang, Danna Yu

**Affiliations:** 1 College of Life Sciences, Zhejiang Normal University, Jinhua 321004, China; 2 Department of Biology, Carleton University, Ottawa, ON K1S5B6, Canada; 3 Key Lab of Wildlife Biotechnology, Conservation and Utilization of Zhejiang Province, Zhejiang Normal University, Jinhua 321004, China

**Keywords:** skink, low-temperature stress, *RT*-qPCR, latitudinal pattern, mitochondrial genome expression

## Abstract

Continual climate change strongly influences temperature conditions worldwide, making ectothermic animals as suitable species for studying the potential impact of climate change on global biodiversity. However, the study of how lizards distributed at different latitudes respond to climate change at the transcriptome level is still insufficient. According to the Climatic Variability Hypothesis (CVH), the range of climate fluctuations experienced by terrestrial animals throughout the year increases with latitude, so individuals at higher latitudes should exhibit greater thermal plasticity to cope with fluctuating environments. Mitochondria, as the energy center of vertebrate cells, may indicate species’ plasticity through the sensitivity of gene expression. In this study, we focused on the changes in transcript levels of liver mitochondrial protein-coding genes (PCGs) in skinks from the genus *Plestiodon* (*P. capito* and *P. elegans*) and the genus *Scincella* (*S. modesta* and *S. reevesii*) under low-temperature conditions of 8 °C, compared to the control group at 25 °C. Species within the same genus of skinks exhibit different latitudinal distribution patterns. We found that the two *Plestiodon* species, *P. elegans* and *P. capito*, employ a metabolic depression strategy (decreased transcript levels) to cope with low temperatures. In contrast, the two *Scincella* species show markedly different patterns: *S. modesta* exhibits significant increases in the transcript levels of six genes (metabolic compensation), while in *S. reevesii*, only two mitochondrial genes are downregulated (metabolic depression) compared to the control group. We also found that *P. capito* and *S. modesta*, which live at mid-to-high latitudes, exhibit stronger adaptive responses and plasticity at the mitochondrial gene level compared to *P. elegans* and *S. reevesii*, which live at lower latitudes. We suggest that this enhanced adaptability corresponds to more significant changes in a greater number of genes (plasticity genes).

## 1. Introduction

Climate change significantly influences global temperature patterns, particularly during milder winters, when the Asian continent frequently experiences notable cooling episodes. This poses a threat to the survival of populations of both aquatic and terrestrial ectotherms. Reptiles are the second-largest group of terrestrial vertebrates after birds [1]. Due to the frequent occurrence of extreme temperatures caused by current global warming, reptile populations worldwide are experiencing a significant decline (www.iucnredlist.org, accessed on 14 June 2024). Temperature fluctuations can disrupt the internal homeostasis of reptiles [2,3,4,5,6]. Unlike endothermic animals, ectothermic animals cannot maintain a constant body temperature and rely on environmental temperatures to regulate their body heat [7]. Environmental temperatures vary due to various factors such as climate gradients along latitudes or altitudes [8]. This variation has different impacts on species based on their sensitivity (or resilience) to temperature change [9,10]. Meanwhile, the response and distribution changes of species due to climate change across geographic gradients are crucial for understanding species biodiversity and conservation [11].

Stevens [12] named the phenomenon where the latitudinal distribution width of animals and plants gradually narrows from high latitudes to low latitudes as Rapoport’s rule. Based on the observation that annual climate variation in tropical regions is relatively lower compared to high latitudes, it is concluded that the physiological thermal range of tropical species should be narrower than that of temperate species. This conclusion has been generalized into the climate variability hypothesis (CVH) [12,13,14,15], which serves as a potential explanation for Rapoport’s rule. This also indicates a positive correlation between a species’ heat tolerance range and the level of climatic variability experienced by the taxa as latitude increases [16]. The CVH has also been validated in other ectotherms, such as insects [17,18], fish [19], amphibians [16], and lizards [20]. Due to their greater thermal safety margins and the flexible regulation of enzyme activity in thermally tolerant species at higher latitudes, their tissues can stay synchronized with their thermal environment [13]. This enables better adaptation to seasonal environmental changes and rapid adjustment to fluctuating conditions. Conversely, tropical animals at lower latitudes exhibit lower physiological plasticity and greater vulnerability to changes in ambient temperatures [14,15,21]. For instance, in studies of lizards of the *Takydromus* genus, a comparison of the thermal adaptation patterns among three lizard species that are distributed along a latitudinal gradient showed that temperate species, such as *T. septentrionalis* and *T. wolteri*, which inhabit higher latitudes, exhibit a greater degree of cold acclimatization compared to tropical species (*T. sexlineatus*). This is reflected in changes in liver size and variations in metabolic intensity and fuel usage [2]. Therefore, a key factor in assessing a species’ vulnerability to climate change is its ability to adapt to temperature changes through plastic and/or evolutionary responses. Based on this background, this study primarily tests whether a species’ capacity to adapt to temperature changes can be reflected through gene expression plasticity.

In cold environments, ectotherms employ a variety of strategies to maintain an optimal body temperature for performance. These include local adaptation (phenotypic changes) [22], spatial movement to match preferred habitats (such as migration) [23], or plastic responses [24]. For example, when habitats are fragmented, hibernating species may be unable to behaviorally adapt to such changes. Therefore, plasticity becomes crucial for species survival under rapidly changing conditions [25,26,27,28]. To some extent, species plasticity depends on historical exposure to different selective pressures [15,29,30]. Both phenotypic plasticity in behavior and physiology may contribute to maintaining internal metabolic balance [2,31,32], but extensive temperature-induced changes in gene expression are also crucial [33,34,35]. Temperature fluctuations can lead to significant changes in gene expression, often involving genes that encode mitochondrial proteins or other proteins with metabolic functions [3,36,37,38,39]. Mitochondria, as the central organelles for ATP production, are increasingly recognized for their significance in species ecology and biogeography [8]. Mitochondrial adaptations involve conservative mechanisms that regulate mitochondrial respiration to maintain cellular and organismal health and survival in dynamic environments. These responses adhere to the general principles of regulatory biology, such as regulation of gene expression, protein synthesis, complex formation, membrane transport, enzyme activity, and regulation of metabolite levels [40]. It is known that cold acclimation triggers metabolic reactions involving increased numbers and capabilities of mitochondria, suggesting that low temperatures might pose a unique challenge in coordinating the functions of nuclear and mitochondrial genomes [41].

The 13 proteins encoded by mitochondrial PCGs are vital constituents of enzymes engaged in both the citric acid cycle and the oxidative phosphorylation (OXPHOS) pathway [42,43,44]. Different populations or species, with varying distributions or habitat use, may experience different selective pressures on mitochondrial genomes. For instance, specific amino acid changes in proteins encoded on the mitochondrial genome, such as cytochrome c oxidase (COX) proteins, have been linked to enhanced mitochondrial respiration under low-oxygen conditions, potentially contributing to animal adaptation to high-altitude environments [45]. Studies of lacertid lizards have shown that climate change has driven selective pressure on the ATP6, ATP8, and ND3 genes. Mutations in ATP and ND genes affect the efficiency of proton transport, balancing the heat produced and ATP synthesis. Simultaneous selection on ATP and ND genes enhances lizards’ adaptability to different climates [46]. Adaptation to different temperatures or other latitude-dependent factors can drive phenotypic and genetic diversity across species [41,47,48,49], which can be observed at the level of gene expression [50,51]. Plasticity in gene expression related to mitochondrial biogenesis and energy metabolism plays a significant role in cold adaptation. For example, at 7 °C, freshwater sticklebacks show upregulation of several genes involved in mitochondrial biogenesis or activity compared to marine sticklebacks, including PPARGC1b and PPARAa [25]. The upregulation of these energy-metabolism-related genes effectively compensates for the reduced oxygen diffusion and metabolic reaction rates in cold environments [34]. In a study by Hong et al. [52], amphibians such as *Dryophytes immaculata*, *Hyla annectans*, and *H. chinensis* frogs, that live at slightly higher latitudes, exhibited strategies to conserve energy and downregulate expression of multiple mitochondrial genes in response to cold stress, thereby demonstrating their adaptation to variable climates in challenging environments. By contrast, a low-latitude dweller *H. zhaopingensis* showed changes in only two mitochondrial genes, seeming to lack sufficient defense mechanisms to cope with cold-induced damage. Hence, differences in mitochondrial gene expression among species at different latitudes in cold environments may potentially indicate species cold tolerance.

**Figure 1:**
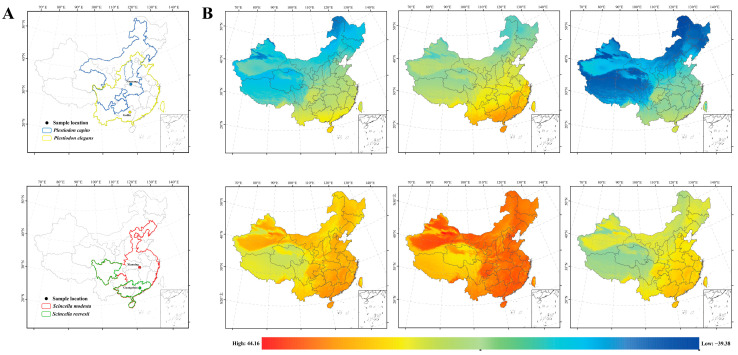
(**A**) Sampling sites and distribution areas of *P. capito*, *P. elegans*, *S. modesta*, and *S. reevesii*. Different colors represent different species. *P. capito* from Nanyang, Henan, is represented by blue (33°08′ N, 112°21′ E). *P. elegans* from Guilin, Guangxi, is represented by yellow (24°18′ N, 109°42′ E). *S. modesta* from Xianning, Hubei, is represented by red (29°34′ N, 114°29′ E). *S. reevesii* from Guangzhou, Guangdong, is represented by green (23°11′ N, 113°23′ E). (**B**) Temperature variation maps for January and July in China. The first row, from left to right, represents the mean temperature, minimum temperature, and maximum temperature in China for January, which is the coldest month of the year. The second row, from left to right, represents the mean temperature, maximum temperature, and minimum temperature in China for July, which is the hottest month of the year. The gradient colors on the map indicate temperature variations in the thermal environment.

Scincidae is the most diverse and widely distributed group among lizard species [53]. The genera *Plestiodon* (blue-tailed skinks) and *Scincella* (ground skinks) are the most prevalent skinks in China. *Plestiodon*, with ten described species [54,55], is primarily located in southern China [56]. Among these, *P. capito* (Gail’s eyelid skink) and *P. elegans* (Shanghai elegant skink) are notable for their wider distributions. *P. capito* is restricted to northern China (Figure 1A), preferring mountainous regions with dense vegetation. Its active season is from late April to early October, becoming active when the daytime temperature is around 25 °C, and only occasionally seen under tree shade when the temperature is above 30 °C [57]. In contrast, *P. elegans* inhabits areas east of a line connecting Hebei to Yunnan on the Chinese mainland [58], including Taiwan (Figure 1A). It thrives in open mountainous areas and is found under rocks in the low mountain forests and along trails south of the Yangtze River, at elevations below 2500 m [59]. As a diurnal lizard living on sunny slopes in mountainous areas, it has a high preferred temperature of 30.4 °C. The genus *Scincella* exhibits particularly high diversity in the tropical and subtropical regions of southern China and Southeast Asia. *S. reevesii* (Reeves’ smooth skink) is only found in the southern parts of China, such as Hainan, Guangxi, Guangdong, and Hong Kong (Figure 1A). And research on its ecological habits and activity temperatures is relatively scarce. *S. modesta* (modest ground skink) is a unique group with a wide distribution that spans both temperate and tropical regions (Figure 1A) (http://www.reptile-database.org/, accessed on 1 September 2024) [60]. The species shows peak activity at temperatures between 23 °C and 25 °C, displays behavior to evade heat when temperatures surpass 28 °C, and enters hibernation when temperatures drop below 8 °C. Their distribution characteristics make them an ideal model for understanding mitochondrial gene-level plasticity in response to climate change under latitudinal gradients. Specifically, we collected *P. capito* from middle-to-high latitudes and *P. elegans* from lower latitudes. As for the *Scincella* genus, we sampled *S. modesta* from mid-to-high latitudes and *S. reevesii* from lower latitudes (Figure 1A). Here, we used 25 °C as the normal temperature and 8 °C to simulate low temperatures for climate simulation, considering that many lizards in China hibernate at 8 °C. Based on the CVH, we predict that lizards from higher latitudes (*P. capito* and *S. modesta*) will tolerate low temperatures better than lizards from lower latitudes (*P. elegans* and *S. reevesii*), which is related to mitochondrial gene expression plasticity. Additionally, by studying the expression changes of 13 mitochondrial PCGs in skinks from different latitudes under cold conditions, we can predict the low-temperature adaptation mechanisms of these skinks and the intrinsic connection between mitochondrial gene expression and cold stress.

## 2. Results

### 2.1. Effect of Cold Exposure on Transcript Levels of Mitochondrial PCGs

**Figure 2:**
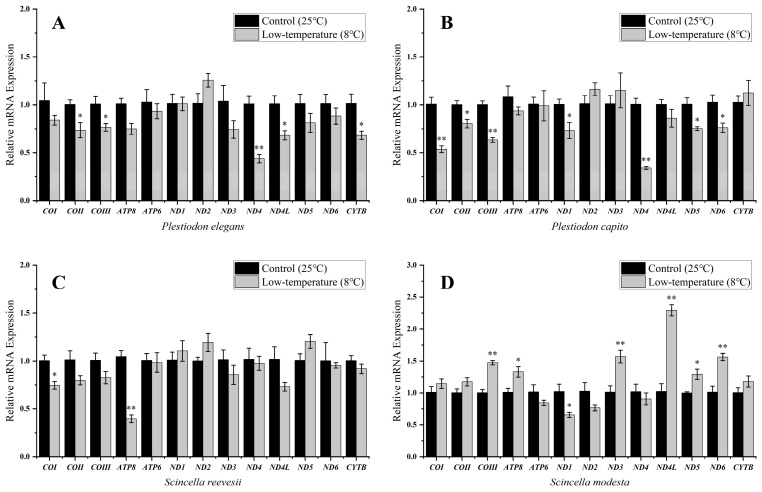
Steady-state transcript levels of 13 PCGs under control (25 °C) and low-temperature (8 °C) stress in (**A**) *Plestiodon elegans*, (**B**) *Plestiodon capito*, (**C**) *Scincella reevesii*, and (**D**) *Scincella modesta*, where “*” indicates a significant difference (*p* < 0.05) and “**” indicates (*p* < 0.01). Gene names are displayed on the *x*-axis and gene steady-state transcript levels are shown on the *y*-axis. The *y*-axis represents mean ± SE. Relative expression levels were normalized using *β-actin* as the reference gene. Specific values can be found in Table S1.

*Plestiodon capito* at mid-to-high latitudes (Table 1) exhibited substantial differences in the expression of seven PCGs with significant downregulation of the transcript levels of COII, ND1, ND5, and ND6 genes (*p* < 0.05), to values of 0.8 ± 0.04, 0.73 ± 0.08, 0.75 ± 0.02, and 0.76 ± 0.05, respectively, as compared with controls. The remaining three PCGs (COI, COIII, and ND4 genes) showed strong significant downregulation in transcript levels (*p* < 0.01), with values of 0.54 ± 0.04, 0.63 ± 0.02, and 0.34 ± 0.01, respectively (Figure 2B). In the low-latitude *P. elegans* (Table 1), the number of downregulated genes decreased, with significant downregulation of the transcript levels of COII, COIII, ND4L, CYTB (*p* < 0.05), and ND4 genes (*p* < 0.01), to values of 0.74 ± 0.08, 0.76 ± 0.04, 0.68 ± 0.05, 0.68 ± 0.04, and 0.44 ± 0.04, respectively, compared with controls (Figure 2A).

**Figure 3:**
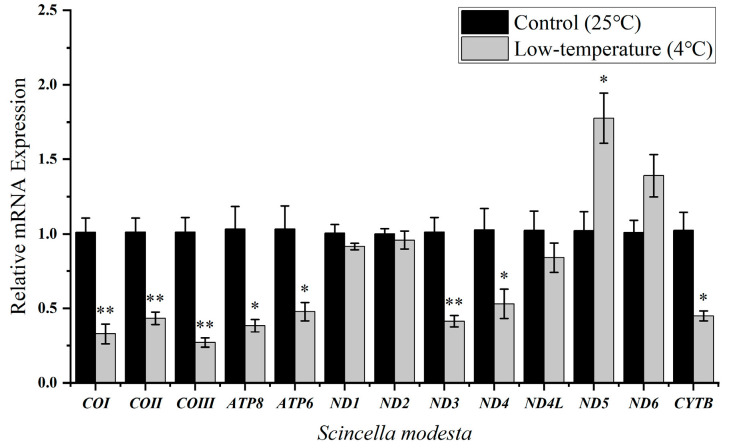
Steady-state transcript levels of 13 PCGs of *Scincella modesta* in response to low-temperature stress. Gene names are displayed on the *x*-axis and gene steady-state transcript levels are shown on the *y*-axis. Asterisks indicate significantly different expression as compared with controls (*, *p* < 0.05) and (**, *p* < 0.01). The *y*-axis represents mean ± SE. Relative expression levels were normalized using *β-actin* as the reference gene. Specific values can be found in Table S1.

Low-temperature treatments had a lesser effect on the transcript levels of mitochondrial genes in the low-latitude skink (Table 1), *S. reevesii,* with significant downregulation in the transcript levels of COI (*p* < 0.05) and ATP8 genes (*p* < 0.01), to 0.75 ± 0.04 and 0.40 ± 0.04, respectively, as compared to the controls (Figure 2C, Table 1). However, *S. modesta* at mid-to-high latitudes (Table 1) showed significant differences in the expression of seven PCGs, with transcript levels of ATP8, ND5 (*p* < 0.05), COIII, ND3, ND4L, and ND6 genes (*p* < 0.01) increasing significantly by 1.33 ± 0.08, 1.29 ± 0.08, 1.48 ± 0.03, 1.57 ± 0.10, 2.29 ± 0.09, and 1.56 ± 0.06 fold, respectively, as compared with controls. However, the transcript level of the ND1 gene showed statistically significant downregulation (*p* < 0.05) with a mean value of 0.65 ± 0.04, as depicted in Figure 2D. To further investigate the opposite mitochondrial gene expression in *S. modesta* at 8 °C, we also conducted stress experiments at 4 °C. Our study demonstrated that as temperature dropped, *S. modesta* experienced an increase in the number of differentially expressed genes, with eight out of nine being downregulated. Notably, the ND5 gene (*p* < 0.01) showed a substantial upregulation, averaging 1.78 ± 0.17 fold. Under 4 °C conditions, cold induction in the liver of the skinks led to substantial reductions in the transcript levels of ND4, ATP6, ATP8, and CYTB genes, with reductions by 0.53 ± 0.10, 0.48 ± 0.06, 0.38 ± 0.04, and 0.45 ± 0.03, respectively, compared to the control group (*p* < 0.05). Furthermore, the analysis revealed highly significant downregulation in the transcript levels of four key PCGs: COI, COII, COIII, and ND3 genes (*p* < 0.01), with mean values of 0.33 ± 0.07, 0.43 ± 0.04, 0.27 ± 0.03, and 0.41 ± 0.04, respectively (Figure 3).

### 2.2. Comparison of the Number of Differentially Expressed Genes

When investigating the *Plestiodon* genus (Table 2), we found that *P. capito* inhabiting higher latitudes exhibited downregulation in the transcript levels of seven mitochondrial genes (*p* < 0.05), with three genes showing very strong down regulation (*p* < 0.01). By contrast, *P. elegans* inhabiting lower latitudes showed downregulation in the transcript levels of five mitochondrial genes (*p* < 0.05) including transcript levels of one gene that displayed a strong significant downregulation (*p* < 0.01). Interestingly, the transcript levels of COII and COIII genes exhibited significant downregulation (*p* < 0.05) in both species. The transcript levels of ND4 gene were also strongly downregulated (*p* < 0.01).

Similar patterns were also observed in the *Scincella* genus (Table 2). We found that in *S. modesta*, inhabiting mid-to-high latitudes, mitochondrial gene plasticity was higher after both 8 °C and 4 °C exposure as compared to *S. reevesii* from lower latitudes. At 8 °C, *S. modesta* showed changes in transcript levels of seven mitochondrial genes (*p* < 0.05), with four genes showing strongly significant upregulation changes (*p* < 0.01). In comparison, *S. reevesii* exhibited downregulation of transcript levels of two mitochondrial genes, with COI (*p* < 0.05), and ATP8 genes showing downregulation (*p* < 0.01). As the temperature dropped to 4 °C, *S. modesta* exhibited a more pronounced downregulation of energy-related mitochondrial genes (eight genes), a pattern similar to that observed in the other three lizards at 8 °C. Both *Scincella* species displayed significant changes in the transcript levels of ATP8 gene, whereas a decrease in COI gene transcript levels was observed in *S. reevesii* exposed to 8 °C and *S. modesta* exposed to 4 °C. Furthermore, compared to the control group, both *P. capito* and *S. modesta*, distributed at mid-to-high latitudes, consistently exhibited a significant reduction in ND1 gene transcripts (*p* < 0.05) in the liver.

## 3. Discussion

### 3.1. Mitochondrial Gene Expression under Low-Temperature Stress

The liver is the primary organ for energy metabolism in organisms and contains abundant mitochondria [61]. Therefore, this study focuses on the liver as the main site for investigating the mitochondrial gene response to low-temperature stress in lizards. Mitochondria serve as the primary energy producers within cells, effectively functioning as the ‘power plants’ by converting food into ATP through oxidative phosphorylation. The optimal performance of this essential function relies on the precise regulation of gene expression within the mitochondrial genome (mtDNA) [62,63]. Our study revealed the sensitivity of mitochondrial genes to temperature variations, with gene expression patterns highlighting both shared characteristics and unique adaptations in the cold tolerance mechanisms observed among skinks from diverse latitudes. These differential responses could be attributed to the distinct temperature tolerances and disparate energy allocation strategies employed by the studied populations.

For terrestrial ectotherms, low-temperature stress is often associated with more severe oxidative damage [64]. In general, *Plestiodon* lizards mainly regulated the expression of cytochrome c oxidase (COX) genes and NADH dehydrogenase (ND) genes to resist the adverse effects of low temperatures. The protein encoded by the COX gene family is a vital component of cytochrome c oxidase (COX), the terminal enzyme in the mitochondrial electron transport chain responsible for oxidative phosphorylation [65]. COX catalyzes the transfer of electrons from reduced cytochrome c to molecular oxygen, a process that combines with the transfer of protons across the inner membrane, thus contributing to the generation of proton gradients [66]. The ND genes constitute different subunits of NADH dehydrogenase (complex I) in the mitochondrial membrane respiratory chain, respectively. They use ubiquinone as an electron acceptor to catalyze the electron transfer of NADH through the respiratory chain, which is critical for the catalytic activity and assembly of complex I [67]. Complex I is one of the main sites for superoxide formation, and the ROS generated by oxidative phosphorylation mainly comes from complex I [68]. The repression of ND gene expression initiates a decline in mitochondrial complex I activity, potentially inducing increased proton leakage and reduced coupling of oxidative phosphorylation. As a consequence, a reduction in the membrane potential difference occurs, diminishing ATP synthesis to mitigate the overproduction of reactive oxygen species [69]. These outcomes align with the documented findings in *Hemidactylus bowringii* [3] and *Sphenomorphus incognitus* [70]. Research investigating liver gene expression in hibernating *Nanorana parkeri* has revealed a notable downregulation of proteins associated with essential metabolic pathways, including mitochondrial oxidative phosphorylation and the respiratory electron transport chain [71]. This downregulation is viewed as a key regulatory mechanism adopted by amphibians during winter hibernation. Given that certain proteins crucial for oxidative phosphorylation and the electron transport chain are encoded by mitochondrial genes [62], the suppression of mitochondrial gene expression significantly contributes to the adaptation of ectothermic species to cold habitats. During the cold winter and spring, when the longest adverse weather occurs and lizards cannot bask or forage, downregulating mitochondrial gene activity is likely an important mechanism to reduce energy expenditure for ectothermic animals preparing for or entering hibernation. This could prolong their survival time in cold environments.

Within the genus *Scincella*, significant disparities were evident between *S. reevesii* and *S. modesta*. The contrasting responses and distinct cold tolerance mechanisms exhibited by the two species under 8 °C low-temperature stress was discernible. However, after a 24 h exposure to 4 °C, the regulation of mitochondrial gene expression in *S. modesta* was similar to that of *S. reevesii* when exposed to 8 °C, with a majority of mitochondrial genes showing downregulation. Interestingly, a noteworthy change in the expression of the ATP8 gene occurred in these two species of *Scincella*. This gene encodes a subunit of ATP synthase on the mitochondrial membrane [72], impacting the rate of ATP generation via the respiratory chain. Variations in ATP8 gene expression levels directly correlate with ATP production rates [73,74,75]. Research has found that in the mitochondrial genome comparison of skinks, the evolution rate of the ATP8 gene is the fastest [76], and climatic conditions promote the selection of this gene, enhancing the adaptability of the lizards in different climates [46]. Mutations in the ATP8 gene may alter mitochondrial performance, increase the production of H_2_O_2_, and affect mitochondrial structure [77]. All cells need to maintain ATP balance, with electron flow toward ATP synthesis, proton leakage (heat generation), or the generation of oxidants within the mitochondria used to maintain metabolic performance and internal balance [78]. The expression of the ATP8 gene may play a coordinating role in this process, helping to alleviate damage caused by cold stress. Further research will help clarify this point. *S. reevesii* might employ a response mechanism akin to *Plestiodon* as discussed previously, primarily regulating metabolic rates through adjustments in COI and ATP8 gene expression levels. The upregulation response of multiple genes in *S. modesta* is not unprecedented in an ectotherm. Low temperatures diminish the flexibility of biomolecules, including DNA, RNA, and proteins, thereby influencing their molecular functions and secondary structures [79]. Upregulation of genes with chaperoning and repair functions (such as protein folding and DNA repair) may indicate a response to the effects of low temperatures [80,81]. As temperatures decrease, ectothermic animals need to allocate additional energy to maintain ATP production and protect cells from oxidative stress [82]. Increased ROS stimulate the activation of heat shock proteins and enhance antioxidant defenses to neutralize free radicals. Many hibernating animals exhibit elevated production of antioxidants, such as superoxide dismutase (SOD), which helps prevent ROS from disrupting protein synthesis [83,84]. Activation of enzymes and maintenance of enzyme activity at low temperatures require substantial energy expenditure. Consequently, the upregulation of mitochondrial multigenes in *S. modesta* at 8 °C may serve as a compensatory metabolic mechanism to balance ATP supply and demand, potentially linked to the observed pseudo-emergence behavior in *S. modesta*. It was observed that during hibernation if there is a short-term temperature increase (above 9 °C), the hibernating *S. modesta* will temporarily emerge, bask for several hours, and then immediately return to its burrow when the temperature drops. Enhancing mitochondrial gene expression can bolster the energy reserves for winter activities in *S. modesta*, mirroring the metabolic adaptation observed in winter-active lizards, which partially compensate for reduced metabolic rates at lower temperatures [85,86,87]. However, under 4 °C stress, *S. modesta* enters deep hibernation, foregoing opportunities for sunbathing and feeding, and instead utilizing reverse compensation to reduce energy expenditure. Increased expression of metabolic genes at low temperatures may counteract reduced reaction rates, aligning with heat compensation. Conversely, decreased expression may indicate metabolic depression, conserving energy during food-limited periods such as winter [41].

### 3.2. The Relationship between Mitochondrial Gene Expressions and the CVH

One approach to assess whether a species may adapt to climate change is to measure evolutionary differences in climate-related traits along latitudinal gradients [88]. Temperature is one of the primary driving factors for all living organisms to adapt to new environments [89,90,91,92]. Studies have found that temperature is one of the determining factors for the current distribution of reptiles in China [93]. In the present study, all samples of *P. elegans* were collected from Guangxi, China, which is a low-latitude region, whereas all samples of *P. capito* were collected from Henan, China, a region of higher latitude. The geographical distribution of *P. capito* extends beyond that of *P. elegans*. The ability of populations with different latitudinal distributions to cope with climate change may be shaped by genetic diversity that arises from long-term exposure to variable environments [13,14,15]. Based on our inference, *P. capito* is likely to have adapted to survive in regions with distinct seasonal climates, implying that this species should exhibit greater plasticity in response to temperature fluctuations. In terrestrial ecosystems, the plasticity of cold tolerance is intrinsically connected to thermal seasonality [94]. In this study, we found that the consistency in mitochondrial gene expression trends under low-temperature conditions between *P. elegans* and *P. capito* suggest a shared response mechanism to cold conditions. Given that the expression of most genes is inherently dynamic, gene expression is plastic and susceptible to both internal and external factors [95]. The significant downregulation of seven genes in *P. capito* suggests that it can regulate more genes as compared to *P. elegans* at low latitudes in order to adapt to environmental temperature changes. When confronted with sudden temperature drops and prolonged cold exposure, *P. capito* can display enhanced plasticity, which is underpinned by a polygenic response. Hong et al. [52] found that *D. immaculata*, distributed in high latitudes, exhibited a significantly greater number of downregulated genes as compared to species in lower latitudes when subjected to cold stress. This finding was consistent with our study and suggested that there were more plasticity genes under low-temperature conditions which may be one of the reasons for the adaptation of reptiles to low temperatures.

In the *Scincella* genus, the liver mitochondrial genome of *S. modesta* from Hubei, China, shows more plasticity genes under low-temperature stress compared to those of *S. reevesii* from Guangdong, China. *S. modesta* inhabits higher latitudes, spanning temperate and tropical zones, whereas *S. reevesii* predominantly resides in tropical and subtropical regions. The latitudinal hypothesis predicts an increasing pattern of thermal plasticity when moving from the equator towards the poles, grounded in the seasonal temperature fluctuations observed [14,96,97]. This anticipated correlation contributes to the heightened vulnerability of tropical organisms to climate change effects [98]. Among the four lizard species investigated, *S. reevesii* exhibits the least significant changes in gene expression, inhabiting the lowest latitudes and demonstrating the poorest cold tolerance plasticity. Extensive evidence underscores the heightened risk posed by extreme climate events to species with limited thermoregulatory capabilities or adapted to narrow climatic niches, particularly tropical ectotherms. Moreover, the geographic distribution of tropical lizards is expected to contract in the future, which further reinforces the high vulnerability of species from low-latitude regions to climate change. The specificity of the changes in gene expression that we observed in *S. modesta* may also be related to its distribution, as it has one of the widest latitudinal distributions among the lizard species studied, ranging from tropical to temperate zones. Transcriptional regulation of cold tolerance in ectotherms exhibits a high degree of geographic variability, and cold tolerance in ectotherms is usually less stable than heat tolerance [99]. Differential cold tolerance mechanisms may result from influences in different distribution areas of ectotherms [3].

Significant similarities and differences in cold response at the mitochondrial gene transcription level can be observed between these two small skinks from the *Scincella* genus. At the same 8 °C cold exposure, most genes in *S. modesta* showed upregulated expression, whereas those in *S. reevesii* were downregulated. Upon exposing *S. modesta* to a lower temperature, a larger number of mitochondrial genes exhibited downregulation compared to *S. reevesii*. Under cold conditions, mitochondrial gene downregulation is commonly associated with hibernation in ectotherms such as amphibians [52,100] and lizards [70]. This suggests that *S. modesta* enter hibernation at a lower temperature than *S. reevesii*, indicating their ability to tolerate lower temperatures. These findings align with the CVH [13,14], which posits that species adapted to wider temperature ranges, like temperate ones, have greater thermal resilience. These populations evolved under more variable climatic conditions and thereby developed more mitochondrial gene expression plasticity. Whether the tolerance to temperature and vulnerability to climate change can be reflected through mitochondrial gene expression, and whether mitochondrial genes can serve as a useful marker for studying reptile temperature stress, remains to be seen with more species included in research.

## 4. Materials and Methods

### 4.1. Sample Collection, Acclimatization, and Low-Temperature Stress

From late April to early May 2023, we captured adult male *Plestiodon elegans* (*n* = 20) from Guilin, Guangxi Province (24°18′ N, 109°42′ E), China, adult male *Plestiodon capito* (*n* = 20) from Nanyang, Henan Province (33°08′ N, 112°21′ E), China, adult male *Scincella reevesii* (*n* = 20) from Guangzhou, Guangdong Province (23°11′ N, 113°23′ E), China, and adult male *Scincella modesta* (*n* = 40) from Xianning, Hubei Province (29°34′ N, 114°29′ E), China (Figure 1, Table 1). These lizards were then acclimated at room temperature (25 °C) in 120 cm × 90 cm × 110 cm plastic incubators for one week in the laboratory. After acclimation, ensuring the health of all lizards, ten individuals were randomly selected for each group. The typical temperature in May is around 25 °C in China, while the hibernation temperature for most lizards is 8 °C. Therefore, 25 °C and 8 °C were used as the control group and experimental group, respectively, with each group treated for 24 h. Due to the unique gene expression pattern observed in *S. modesta* at 8 °C during analysis of our results, we conducted an additional experiment. We added 20 more individuals of *S. modesta*, with one group of 10 individuals exposed to a lower temperature (4 °C) for 24 h, and another 10 kept at 25 °C as a control group, to further investigate its cold tolerance regulatory mechanisms.

### 4.2. DNA Extraction and Sequencing

Although the mitochondrial genome sequences of these four lizard species have been reported online, we re-sequenced the mitochondrial genomes of the four species collected from different locations to ensure the accuracy of our *RT*-qPCR results and minimize potential confounding effects due to inter-specific differences. Genomic DNA was extracted from tail tissue tips, following the manufacturer’s protocol using the Ezup Column Animal Genomic DNA Purification Kit (Sangon Biotech Company, Shanghai, China). The extracted DNA was then separated via 1% agarose gel electrophoresis. In this study, the mitochondrial genome sequence of *S. reevesii* was obtained using Sanger sequencing, whereas the remaining three lizard species were sequenced using the NGS method described above. We employed the lizard primer set originally designed by Kumazawa [101] with modifications, altering eleven primer pairs (as detailed in Table S2) to target various sub-segments. To fill in the remaining sequence gaps, we utilized Primer Premier 5.0 software [102] for the design of specific primer sets, ensuring comprehensive amplification. Samples with DNA extraction concentrations exceeding 25 μg/mL were sent to BGI (Shenzhen, China) for next-generation sequencing (NGS). Genome DNA sequencing was conducted on the Illumina HiSeq 2000 (Illumina, Inc., San Diego, CA, USA.) platform with 150 bp paired-end reads. Following quality assessment of the raw sequencing data using fastQC v.0.11.6, the clean data was used for genome assembly.

### 4.3. Mitochondrial Genome Assembly and Annotation

The mitochondrial genome sequences of *P. elegans*, *P. capito*, and *S. modesta* were reconstructed from the returned data by employing NOVOPlasty v.4.2 [103] and GetOrganelle v.1.7.1 [104]. Seqman in DNASTAR v.6.0 was utilized to align the *S. reevesii* sequencing results, followed by manual verification through Sanger sequencing and subsequent assembly [105]. The tRNA positions were sourced from the Galaxy Europe v 23.1 platform (https://usegalaxy.eu/, accessed on 27 November 2023). To manually annotate and position the 13 PCGs, two rRNAs, and the control region of *P. elegans*, *P. capito*, *S. modesta*, and *S. reevesii*, as per reference sequences with accession numbers KM508815, AB183287, MW327509, and MN832615 retrieved from the NCBI, respectively, we utilized Mega 7.0 [106] in conjunction with SnapGene Viewer v.6.2.2 (http://www.snapgene.com/, accessed on 27 November 2023).

### 4.4. Tissue Source, RNA Extraction and cDNA Synthesis

We selected four individuals of *P. elegans*, *P. capito*, *S. reevesii*, and *S. modesta* from each of the two experimental conditions—ambient control conditions at 25 °C and cold-acclimated conditions at 8 °C. Each temperature treatment group was randomly chosen for the experiment. Due to the relatively low sensitivity of the central nervous system to hypoxia in reptiles, euthanasia using decapitation was performed on them. These individuals were then placed on a chilled dissection tray and dissected [3], and liver tissue was immediately collected and placed into RNA-free 1.5 mL tubes. Liver samples were promptly cryopreserved by immersion in liquid nitrogen and subsequently stored in an ultra-low-temperature freezer at −80 °C. Total RNA was extracted and purified from 32 liver samples using the Animal Tissue Total RNA Extraction Kit (Forgene Company, Chengdu, China). Subsequently, the samples underwent electrophoresis on a 1% agarose gel at 120 V and 120 mA for 20 min and were stained with Goldview (10,000×). Clear bands of 28S and 18S ribosomal RNA confirmed RNA integrity. Due to the potential interference of genomic DNA on the results, we treated the extracted RNA samples using the PrimeScript™ RT Reagent Kit (including gDNA Eraser and PrimeScript™ RT Master Mix) (Takara, Japan) at 42 °C for 2 min to remove genomic DNA. The RNA was then reverse-transcribed into cDNA, with the reaction carried out under the following PCR parameters: 37 °C 15 min, 85 °C 5 s, 4 °C.

### 4.5. RT-qPCR Primer Design and Reaction

Based on the mitochondrial gene sequences obtained for *P. elegans*, *P. capito*, *S reevesii*, and *S. modesta*, we utilized Primer Premier 6.0 software (http://www.premierbiosoft.com, accessed on 1 December 2023) to design *RT*-qPCR primers. *β-actin* was employed as the reference gene [107] because no significant differences were seen in the gene expression of *β-actin* across different temperatures. The upstream primer sequence for *β-actin* amplification was GATCTGGCATCACACTTTCT, and the downstream primer was GTGACACCATCACCAGA [108]. Primers were selected based on *RT*-qPCR reactions as depicted in Table S3. Three technical replicates were employed to assess the gene expression corresponding to each primer pair. The StepOnePlus™ Real-Time PCR System, manufactured by Life Technologies (Carlsbad, CA, USA), was utilized for the quantification of transcript levels of the 13 PCGs. Each sample’s reaction mixture contained 10 µL of SYBR Premix Ex Taq II (2×), 0.4 µL of ROX Reference Dye (50×), 0.8 µL of forward and reverse primers (10 µM), 6 µL of ddH_2_O, and 2 µL of RT reaction mixture (cDNA). The extension stage was set to collect fluorescence signals. After amplification, a melting curve analysis was used to determine the specificity of the amplification products. The temperature increased slowly from 60 °C to 95 °C, continuously measuring the fluorescence intensity of the samples in order to obtain the melting curve. The process included an initial denaturation step at 95 °C for 30 s, followed by 40 cycles of 5 s at 95 °C, and 30 s at 55 °C.

### 4.6. Data Analysis

Fluorescence quantitative experiments and StepOnePlus™ Real-Time PCR System (Applied Biosystems, Foster City, CA, USA) were utilized to measure the transcript levels of the 13 mitochondrial PCGs. The Cycle Threshold (Ct) values for each sample were determined, where Ct values indicate the number of cycles needed for the fluorescent signal to reach a predefined threshold. Each Ct value was linearly correlated with the logarithm of the initial copy number of the respective template. Expression of each gene was calculated as 2^−ΔΔCt^ (ΔCt = Ct_target gene_ − Ct_reference gene_). In this study, each group consisted of four independent biological replicates, and the results were reported as mean ± SE (standard error). Statistical analyses were conducted to assess differences between the values using independent sample *t*-tests, as implemented in SPSS 21.0 (SPSS, Inc., Chicago, IL, USA). Significance was determined using a threshold of *p* < 0.05, indicating that values below this threshold were statistically significant [109]. Origin 8.0 [110] was used to plot the transcript levels of the obtained 13 mitochondrial PCGs, facilitating a clearer comparison of gene expression levels.

## 5. Conclusions

Temperature has a significant impact on the distribution of ectotherms, and the current severity of climate change on Earth may lead to the extinction of selected animal species, with the degree of impact partly dependent on their plasticity to climate change. Populations that evolved under more variable climatic conditions generally exhibit broader thermal tolerance but also exhibit more mitochondrial gene expression plasticity. Here, we found that, compared to lizards from mid-to-high latitudes, species from low latitudes have fewer plasticity genes when facing low-temperature environments, making them more susceptible to climate change, which supports the CVH. It is noteworthy that *S. modesta* exhibited a metabolic compensation mechanism at 8 °C during winter, whereas the other three species showed a metabolic depression strategy where gene expression decreased. Increasing gene expression may provide energy for *S. modesta* to sustain winter activities. Furthermore, given the sensitivity of lizard mitochondrial genes to cold stress, their expression patterns may serve as indicators of vulnerability when organisms face temperature changes.

## Figures and Tables

**Table 1:** Collection information about samples used in this study and their NCBI GenBank accession numbers.

Collection Time	Species	Sex	Latitude (N)	Longitude (E)	Altitude	Locality	Accession No.
Spring	*Plestiodon elegans*	male	24°18′	109°42′	202 m	Guilin, Guangxi	PP946410
Spring	*Plestiodon capito*	male	33°08′	112°21′	203 m	Nanyang, Henan	PP946409
Spring	*Scincella reevesii*	male	23°11′	113°23′	204 m	Guangzhou, Guangdong	PP946408
Spring	*Scincella modesta*	male	29°34′	114°29′	218 m	Xianning, Hubei	PP946411

**Table 2:** The genes that are differentially expressed and co-expressed in the four species under low-temperature conditions. ‘+’ indicates upregulation, ‘−’ indicates downregulation, and blank indicates no significant change in gene expression for that particular species.

Group	Gene												
	*COI*	*COII*	*COIII*	*ATP*8	*ATP*6	*ND*1	*ND*2	*ND*3	*ND*4	*ND*4*L*	*ND*5	*ND*6	*CYTB*
*S. modesta* (4 °C)	−	−	−	−	−			−	−		+		−
*S. modesta* (8 °C)			+	+		−		+		+	+	+	
*S. reevesii* (8 °C)	−			−									
*P. elegans* (8 °C)		−	−						−	−			−
*P. capito* (8 °C)	−	−	−			−			−		−	−	

## Data Availability

Data to support this study are available from the National Center for Biotechnology Information (https://www.ncbi.nlm.nih.gov, accessed on 25 June 2024). The GenBank numbers are PP946408- PP946411.

## Supplementary Materials

The following supporting information can be downloaded at: https://www.mdpi.com/article/10.3390/ijms251910637/s1.

## References

[1] Pincheira Donoso D., Bauer A.M., Meiri S., Uetz P. (2013). Global taxonomic diversity of living reptiles. PLoS ONE.

[2] Sun B.J., Williams C.M., Li T., Speakman J.R., Jin Z.G., Lu H.L., Luo L.G., Du W.G. (2022). Higher metabolic plasticity in temperate compared to tropical lizards suggests increased resilience to climate change. Ecol. Monogr..

[3] He J.Y., Zhan L.M., Meng S.Q., Wang Z., Gao L.L., Wang W.J., Storey K.B., Zhang Y.P., Yu D.N. (2024). Differential mitochondrial genome expression of three sympatric lizards in response to low-temperature stress. Animals.

[4] Sun B.J., Li T., Gao J., Ma L., Du W.G. (2015). High incubation temperatures enhance mitochondrial energy metabolism in reptile embryos. Sci. Rep..

[5] Ding Z.H., Wang X.F., Zou T.T., Hao X., Zhang Q., Sun B.J., Du W.G. (2024). Climate warming has divergent physiological impacts on sympatric lizards. Sci. Total Environ..

[6] Stroud J.T., Mothes C.C., Beckles W., Heathcote R.J.P., Donihue C.M., Losos J.B. (2020). An extreme cold event leads to community-wide convergence in lower temperature tolerance in a lizard community. Biol. Lett..

[7] Burraco P., Orizaola G., Monaghan P., Metcalfe N.B. (2020). Climate change and ageing in ectotherms. Glob. Change Biol..

[8] Sokolova I.M. (2023). Ectotherm mitochondrial economy and responses to global warming. Acta Physiol..

[9] Huey R.B., Kearney M.R., Krockenberger A., Holtum J.A., Jess M., Williams S.E. (2012). Predicting organismal vulnerability to climate warming: Roles of behaviour, physiology and adaptation. Phil. Trans. R. Soc. Lond. B.

[10] Williams S.E., Shoo L.P., Isaac J.L., Hoffmann A.A., Langham G. (2008). Towards an integrated framework for assessing the vulnerability of species to climate change. PLoS Biol..

[11] Bonebrake T.C., Brown C.J., Bell J.D., Blanchard J.L., Chauvenet A., Champion C., Chen I.C., Clark T.D., Colwell R.K., Danielsen F. (2018). Managing consequences of climate-driven species redistribution requires integration of ecology, conservation and social science. Biol. Rev..

[12] Stevens G.C. (1989). The latitudinal gradient in geographical range: How so many species coexist in the tropics. Am. Nat..

[13] Deutsch C.A., Tewksbury J.J., Huey R.B., Sheldon K.S., Ghalambor C.K., Haak D.C., Martin P.R. (2008). Impacts of climate warming on terrestrial ectotherms across latitude. Proc. Natl. Acad. Sci. USA.

[14] Ghalambor C.K., Huey R.B., Martin P.R., Tewksbury J.J., Wang G. (2006). Are mountain passes higher in the tropics? Janzen’s hypothesis revisited. Integr. Comp. Biol..

[15] Tewksbury J.J., Huey R.B., Deutsch C.A. (2008). Putting the heat on tropical animals. Science.

[16] Gutiérrez-Pesquera L.M., Tejedo M., Olalla-Tárraga M.Á., Duarte H., Nicieza A., Solé M. (2016). Testing the climate variability hypothesis in thermal tolerance limits of tropical and temperate tadpoles. J. Biogeogr..

[17] Addo-Bediako A., Chown S.L., Gaston K.J. (2000). Thermal tolerance, climatic variability and latitude. Proc. R. Soc. Lond. B Biol. Sci..

[18] Calosi P., Bilton D.T., Spicer J.I., Votier S.C., Atfield A. (2010). What determines a species’ geographical range? Thermal biology and latitudinal range size relationships in European diving beetles (Coleoptera: Dytiscidae). Anim. Ecol..

[19] Smith S., Brauer C.J., Sasaki M., Unmack P.J., Guillot G., Laporte M., Bernatchez L., Beheregaray L.B. (2020). Latitudinal variation in climate-associated genes imperils range edge populations. Mol. Ecol..

[20] Clusella-Trullas S., Blackburn T.M., Chown S.L. (2011). Climatic predictors of temperature performance curve parameters in ectotherms imply complex responses to climate change. Am. Nat..

[21] Overgaard J., Kristensen T.N., Mitchell K.A., Hoffmann A.A. (2011). Thermal tolerance in widespread and tropical *Drosophila* species: Does phenotypic plasticity increase with latitude?. Am. Nat..

[22] Parmesan C. (2006). Ecological and evolutionary responses to recent climate change. Annu. Rev. Ecol. Evol. Syst..

[23] Pecl G.T., Araújo M.B., Bell J.D., Blanchard J., Bonebrake T.C., Chen I.C., Clark T.D., Colwell R.K., Danielsen F., Evengård B. (2017). Biodiversity redistribution under climate change: Impacts on ecosystems and human well-being. Science.

[24] Davis M.B., Shaw R.G., Etterson J.R. (2005). Evolutionary responses to changing climate. Ecology.

[25] Morris M.R., Richard R., Leder E.H., Barrett R.D., Aubin-Horth N., Rogers S.M. (2014). Gene expression plasticity evolves in response to colonization of freshwater lakes in threespine stickleback. Mol. Ecol..

[26] Teplitsky C., Mills J.A., Alho J.S., Yarrall J.W., Merilä J. (2008). Bergmann’s rule and climate change revisited: Disentangling environmental and genetic responses in a wild bird population. Proc. Natl. Acad. Sci. USA.

[27] Einum S., Burton T. (2023). Divergence in rates of phenotypic plasticity among ectotherms. Biol. Lett..

[28] Aguilar-Kirigin A.J., Naya D.E. (2013). Latitudinal patterns in phenotypic plasticity: The case of seasonal flexibility in lizards’ fat body size. Oecologia.

[29] Gotthard K., Nylin S. (1995). Adaptive plasticity and plasticity as an adaptation: A selective review of plasticity in animal morphology and life history. Oikos.

[30] Logan M.L., Cox C.L. (2020). Genetic constraints, transcriptome plasticity, and the evolutionary response to climate change. Front. Genet..

[31] Kammer A.R., Orczewska J.I., O’Brien K.M. (2011). Oxidative stress is transient and tissue specific during cold acclimation of threespine stickleback. J. Exp. Biol..

[32] Kelly S.A., Panhuis T.M., Stoehr A.M. (2011). Phenotypic plasticity: Molecular mechanisms and adaptive significance. Compr. Physiol..

[33] West-Eberhard M.J. (2003). Developmental Plasticity and Evolution.

[34] Guderley H. (2004). Locomotor performance and muscle metabolic capacities: Impact of temperature and energetic status. Comp. Biochem. Physiol..

[35] Podrabsky J.E., Somero G.N. (2004). Changes in gene expression associated with acclimation to constant temperatures and fluctuating daily temperatures in an annual killifish *Austrofundulus limnaeus*. J. Exp. Biol..

[36] Itoi S., Kinoshita S., Kikuchi K., Watabe S. (2003). Changes of carp F_o_F_1_-ATPase in association with temperature acclimation. Am. J. Physiol. Integr. Comp. Physiol..

[37] Gracey A.Y., Fraser E.J., Li W., Fang Y., Taylor R.R., Rogers J., Brass A., Cossins A.R. (2004). Coping with cold: An integrative, multitissue analysis of the transcriptome of a poikilothermic vertebrate. Proc. Natl. Acad. Sci. USA.

[38] Vornanen M., Hassinen M., Koskinen H., Krasnov A. (2005). Steady-state effects of temperature acclimation on the transcriptome of the rainbow trout heart. Am. J. Physiol. Regul. Integr. Comp. Physiol..

[39] Chou M.Y., Hsiao C.D., Chen S.C., Chen I.W., Liu S.T., Hwang P.P. (2008). Effects of hypothermia on gene expression in zebrafish gills: Upregulation in differentiation and function of ionocytes as compensatory responses. J. Exp. Biol..

[40] Bennett C.F., Latorre-Muro P., Puigserver P. (2022). Mechanisms of mitochondrial respiratory adaptation. Nat. Rev. Mol. Cell Biol..

[41] Healy T.M., Bryant H.J., Schulte P.M. (2017). Mitochondrial genotype and phenotypic plasticity of gene expression in response to cold acclimation in killifish. Mol. Ecol..

[42] Ballard J.W.O., Pichaud N. (2014). Mitochondrial DNA: More than an evolutionary bystander. Funct. Ecol..

[43] Chong R.A., Mueller R.L. (2013). Low metabolic rates in salamanders are correlated with weak selective constraints on mitochondrial genes. Evolution.

[44] Zhang K., Sun J., Xu T., Qiu J.W., Qian P.Y. (2021). Phylogenetic relationships and adaptation in deep-sea mussels: Insights from mitochondrial genomes. Int. J. Mol. Sci..

[45] Luo Y.J., Gao W.X., Gao Y.Q., Tang S., Huang Q.Y., Tan X.L., Chen J., Huang T. (2008). Mitochondrial genome analysis of *Ochotona curzoniae* and implication of cytochrome c oxidase in hypoxic adaptation. Mitochondrion.

[46] Zhang X., Chen J., Luo H.Y., Chen X., Zhong J., Ji X. (2024). Climate-driven mitochondrial selection in lacertid lizards. Ecol. Evol..

[47] Powers D.A., Smith M., Gonzalez Villasenor I., DiMichele L. (1993). A Multidisciplinary Approach to the Selectionist/Neutralist Controversy Using the Model Teleost, Fundulus heteroclitus.

[48] Powers D.A., Schulte P.M. (1998). Evolutionary adaptations of gene structure and expression in natural populations in relation to a changing environment: A multidisciplinary approach to address the million-year saga of a small fish. J. Exp. Zool..

[49] Schulte P.M. (2001). Environmental adaptations as windows on molecular evolution. Comp. Biochem. Physiol. B Biochem. Mol. Biol..

[50] Whitehead A., Crawford D.L. (2006). Neutral and adaptive variation in gene expression. Proc. Natl. Acad. Sci. USA.

[51] Dayan D.I., Crawford D.L., Oleksiak M.F. (2015). Phenotypic plasticity in gene expression contributes to divergence of locally adapted populations of *Fundulus heteroclitus*. Mol. Ecol..

[52] Hong Y.H., Yuan Y.N., Li K., Storey K.B., Zhang J.Y., Zhang S.S., Yu D.N. (2024). Differential mitochondrial genome expression of four Hylid frog species under low-temperature stress and its relationship with Amphibian temperature adaptation. Int. J. Mol. Sci..

[53] Hedges S.B. (2014). The high-level classification of skinks (Reptilia, Squamata, Scincomorpha). Zootaxa.

[54] Cai B., Wang Y.Z., Chen Y.Y., Li J. (2015). A revised taxonomy for Chinese reptiles. Biodivers. Sci..

[55] Kurita K., Ota H., Hikida T. (2017). A new species of *Plestiodon* (Squamata: Scincidae) from the Senkaku Group, Ryukyu Archipelago, Japan. Zootaxa.

[56] Brandley M.C., Ota H., Hikida T., de Oca A.N.M., Feria-Ortiz M., Guo X., Wang Y. (2012). The phylogenetic systematics of blue-tailed skinks (Plestiodon) and the family Scincidae. Zool. J. Linn. Soc..

[57] Jiang Y.F. (2005). A study on habit of *Eumeces capito*. Sichuan J. Zool..

[58] Pope C.H., Granger W. (1929). Notes on Reptiles from Fukien and Other Chinese Provinces. Bull. AMNH.

[59] Norval G., Huang S.-C., Mao J.-J., Goldberg S.R. (2012). Notes on some dietary items of *Eutropis longicaudata* (Hallowell, 1857), *Japalura polygonata* xanthostoma Ota, 1991, *Plestiodon elegans* (Boulenger, 1887), and *Sphenomorphus indicus* (Gray, 1853) from Taiwan. Herpetol. Notes.

[60] Uetz P., Koo M.S., Aguilar R., Brings E., Catenazzi A., Chang A.T., Wake D. (2021). A quarter century of reptile and amphibian databases. Herpetol. Rev.

[61] Zang X.Y., Guo J.L., Geng X.F., Li P.F., Sun J.Y., Wang Q.W., Xu C.S. (2016). Proteome analysis of the liver in the Chinese fire-bellied newt *Cynops orientalis*. Genet. Mol. Res.

[62] Wu Z., Sainz A.G., Shadel G.S. (2021). Mitochondrial DNA: Cellular genotoxic stress sentinel. Trends Biochem. Sci..

[63] Gustafsson C.M., Falkenberg M., Larsson N.-G. (2016). Maintenance and expression of mammalian mitochondrial DNA. Annu. Rev. Biochem..

[64] Ritchie D.J., Friesen C.R. (2022). Invited review: Thermal effects on oxidative stress in vertebrate ectotherms. Comp. Biochem. Physiol. A Mol. Integr. Physiol..

[65] Zhang K.Z., Wang G.H., Zhang X.B., Huttemann P.P., Qiu Y., Liu J., Mitchell A., Lee I., Zhang C., Lee J.S. (2016). COX7AR is a Stress-inducible Mitochondrial COX Subunit that Promotes Breast Cancer Malignancy. Sci. Rep..

[66] Timon-Gomez A., Nyvltova E., Abriata L.A., Vila A.J., Hosler J., Barrientos A. (2018). Mitochondrial cytochrome c oxidase biogenesis: Recent developments. Semin. Cell Dev. Biol..

[67] Formosa L.E., Dibley M.G., Stroud D.A., Ryan M.T. (2018). Building a complex complex: Assembly of mitochondrial respiratory chain complex I. Semin. Cell Dev. Biol..

[68] Barbhuiya P.A., Uddin A., Chakraborty S. (2021). Codon usage pattern and evolutionary forces of mitochondrial ND genes among orders of class Amphibia. J. Cell. Physiol..

[69] Romshin A.M., Osypov A.A., Popova I.Y., Zeeb V.E., Sinogeykin A.G., Vlasov I.I. (2022). Heat release by isolated mouse brain mitochondria detected with diamond thermometer. Nanomaterials.

[70] Zhan L.M., He J.Y., Meng S.Q., Guo Z.Q., Chen Y.X., Storey K.B., Zhang J.Y., Yu D.N. (2024). Mitochondrial protein-coding gene expression in the lizard *Sphenomorphus incognitus* (Squamata: Scincidae) responding to different temperature stresses. Animals.

[71] Niu Y.G., Wei D.B., Zhang X.J., Xu T.S., Li X.Y., Zhang H.Y., An Z.F., Kenneth B.S., Chen Q. (2024). Surviving winter on the Qinghai-Xizang Plateau: Extensive reversible protein phosphorylation plays a dominant role in regulating hypometabolism in hibernating *Nanorana parkeri*. Zool. Res..

[72] Rak M., Su C.H., Xu J.T., Azpiroz R., Singh A.M., Tzagoloff A. (2016). Regulation of mitochondrial translation of the *ATP8*/*ATP6* mRNA by Smt1p. Mol. Biol. Cell.

[73] Kagawa Y., Hamamoto T., Endo H., Ichida M., Shibui H., Hayakawa M. (1997). Genes of human ATP synthase: Their roles in physiology and aging. Biosci. Rep..

[74] Zhang Q.L., Zhang L., Zhao T.X., Wang J., Zhu Q.H., Chen J.Y., Yuan M.L. (2017). Gene sequence variations and expression patterns of mitochondrial genes are associated with the adaptive evolution of two *Gynaephora* species (Lepidoptera: Lymantriinae) living in different high-elevation environments. Gene.

[75] Cizkova A., Stranecky V., Ivanek R., Hartmannova H., Noskova L., Piherova L., Tesarova M., Hansikova H., Honzik T., Zeman J. (2008). Development of a human mitochondrial oligonucleotide microarray (h-MitoArray) and gene expression analysis of fibroblast cell lines from 13 patients with isolated F_1_F_0_ ATP synthase deficiency. BMC Genom..

[76] Chen L., Lin Y., Xiao Q., Lin Y., Du Y., Lin C., Ward-Fear G., Hu C., Qu Y., Li H. (2021). Characterization of the complete mitochondrial genome of the many-lined sun skink (*Eutropis multifasciata*) and comparison with other Scincomorpha species. Genomics.

[77] Yu X., Wester-Rosenlöf L., Gimsa U., Holzhueter S.-A., Marques A., Jonas L., Hagenow K., Kunz M., Nizze H., Tiedge M. (2009). The mtDNA nt7778 G/T polymorphism affects autoimmune diseases and reproductive performance in the mouse. Hum. Mol. Genet..

[78] Brown J.A., Sammy M.J., Ballinger S.W. (2020). An evolutionary, or “Mitocentric” perspective on cellular function and disease. Redox Biol..

[79] Willmer P. (2002). Biochemical adaptation-Mechanism and process in physiological evolution. Science.

[80] Todgham A.E., Hoaglund E.A., Hofmann G.E. (2007). Is cold the new hot? Elevated ubiquitin-conjugated protein levels in tissues of Antarctic fish as evidence for cold-denaturation of proteins in vivo. J. Comp. Physiol. B.

[81] Feiner N., Rago A., While G.M., Uller T. (2018). Developmental plasticity in reptiles: Insights from temperature-dependent gene expression in wall lizard embryos. J. Exp. Zool. A Ecol. Integr. Physiol..

[82] Bury S., Cichoń M., Bauchinger U., Sadowska E.T. (2018). High oxidative stress despite low energy metabolism and vice versa: Insights through temperature acclimation in an ectotherm. J. Therm. Biol.

[83] Allan M.E., Storey K.B. (2012). Expression of NF-κB and downstream antioxidant genes in skeletal muscle of hibernating ground squirrels, *Spermophilus tridecemlineatus*. Cell Biochem. Funct..

[84] Vucetic M., Stancic A., Otasevic V., Jankovic A., Korac A., Markelic M., Velickovic K., Golic I., Buzadzic B., Storey K.B. (2013). The impact of cold acclimation and hibernation on antioxidant defenses in the ground squirrel (*Spermophilus citellus*): An update. Free Radical Biol. Med..

[85] Zari T.A. (2013). Seasonal acclimation in resting metabolism of the skink, *Mabuya brevicollis* (Reptilia: Scincidae) from southwestern Saudi Arabia. J. Therm. Biol..

[86] Gregory S.A. (1982). Biology of the Reptilia.

[87] Christian K.A., Conley K.E. (1994). Activity and resting metabolism of varanid lizards compared with ‘typical’ lizards. Aust. J. Zool..

[88] Umina P.A., Weeks A.R., Kearney M.R., McKechnie S.W., Hoffmann A.A. (2005). A rapid shift in a classic clinal pattern in *Drosophila* reflecting climate change. Science.

[89] Powers D.A., Lauerman T., Crawford D., DiMichele L. (1991). Genetic mechanisms for adapting to a changing environment. Annu. Rev. Genet..

[90] Somero G. (2010). The physiology of climate change: How potentials for acclimatization and genetic adaptation will determine ‘winners’ and ‘losers’. J. Exp. Biol..

[91] Hoffmann A.A., Sgrò C.M. (2011). Climate change and evolutionary adaptation. Nature.

[92] Amarasekare P., Savage V. (2012). A framework for elucidating the temperature dependence of fitness. Am. Nat..

[93] Mi C.R., Han X.Z., Jiang Z.W., Zeng Z.G., Du W.G., Sun B.J. (2024). Precipitation and temperature primarily determine the reptile distributions in China. Ecography.

[94] Gunderson A.R., Stillman J.H. (2015). Plasticity in thermal tolerance has limited potential to buffer ectotherms from global warming. Proc. R. Soc. B.

[95] Rivera H.E., Aichelman H.E., Fifer J.E., Kriefall N.G., Wuitchik D.M., Wuitchik S.J.S., Davies S.W. (2021). A framework for understanding gene expression plasticity and its influence on stress tolerance. Ecol. Evol..

[96] Chown S., Gaston K., Robinson D. (2004). Macrophysiology: Large-Scale Patterns in Physiological Traits and Their Ecological Implications.

[97] Bozinovic F., Calosi P., Spicer J.I. (2011). Physiological correlates of geographic range in animals. Annu. Rev. Ecol. Evol. Syst..

[98] Huey R.B., Deutsch C.A., Tewksbury J.J., Vitt L.J., Hertz P.E., Álvarez Pérez H.J., Garland T. (2009). Why tropical forest lizards are vulnerable to climate warming. Proc. R. Soc. B.

[99] Lancaster L.T., Dudaniec R.Y., Chauhan P., Wellenreuther M., Svensson E.I., Hansson B. (2016). Gene expression under thermal stress varies across a geographical range expansion front. Ecol. Evol..

[100] Wang J.Y., Zhang L.H., Hong Y.H., Cai L.N., Storey K.B., Zhang J.Y., Zhang S.S., Yu D.N. (2023). How does mitochondrial protein-coding gene expression in *Fejervarya kawamurai* (Anura: Dicroglossidae) respond to extreme temperatures?. Animals.

[101] Kumazawa Y., Endo H. (2004). Mitochondrial genome of the Komodo dragon: Efficient sequencing method with reptile-oriented primers and novel gene rearrangements. DNA Res..

[102] Lalitha S. (2000). Primer Premier 5. Biotech. Softw. Internet Rep..

[103] Dierckxsens N., Mardulyn P., Smits G. (2017). NOVOPlasty: De novo assembly of organelle genomes from whole genome data. Nucleic Acids Res..

[104] Jin J.J., Yu W.B., Yang J.B., Song Y., DePamphilis C.W., Yi T.S., Li D.Z. (2020). GetOrganelle: A fast and versatile toolkit for accurate de novo assembly of organelle genomes. Genome Biol..

[105] Burland T.G. (1999). DNASTAR’s Lasergene sequence analysis software. Bioinformatics Methods and Protocols.

[106] Kumar S., Stecher G., Tamura K. (2016). MEGA7: Molecular evolutionary genetics analysis version 7.0 for bigger datasets. Mol. Biol. Evol..

[107] Biederman J., Yee J., Cortes P. (2004). Validation of internal control genes for gene expression analysis in diabetic glomerulosclerosis. Kidney Int..

[108] Cai L.N., Zhang L.H., Lin Y.J., Wang J.Y., Storey K.B., Zhang J.Y., Yu D.N. (2023). Two-fold *ND5* genes, three-fold control regions, incRNA, and the “missing” *ATP8* found in the mitogenomes of *Polypedates megacephalus* (Rhacophridae: *Polypedates*). Animals.

[109] Moeller A.H., Ivey K., Cornwall M.B., Herr K., Rede J., Taylor E.N., Gunderson A.R. (2020). The lizard gut microbiome changes with temperature and is associated with heat tolerance. App. Environ. Microb..

[110] May R.A., Stevenson K.J. (2009). Software review of Origin 8. J. Am. Chem. Soc..

