# Supplementary material for: Comparison of Mitochondrial Genome Expression Differences among Four Skink Species Distributed at Different Latitudes under Low-Temperature Stress
Source: Int J Mol Sci. 2024 Oct 2;25(19):10637. doi: 10.3390/ijms251910637 (PMC11605214; doi:10.3390/ijms251910637)
Supplement: Supplementary file 1 [file ijms-25-10637-s001.zip › Table S1.pdf]

Gene	Average_2^-ΔΔCT_Control	SE_Control	Average_2^-ΔΔCT_treatment	SE_treatment	*P* value
			Plestiodon elegans (8°C)		
COI	1.044	0.185	0.840	0.051	0.366
COII	1.003	0.048	0.735	0.079	0.034*
COIII	1.009	0.079	0.765	0.040	0.045*
ATP8	1.011	0.056	0.748	0.057	0.052
ATP6	1.028	0.133	0.933	0.078	0.566
ND1	1.014	0.097	1.009	0.071	0.970
ND2	1.016	0.101	1.258	0.071	0.198
ND3	1.016	0.165	0.743	0.092	0.147
ND4	1.010	0.080	0.438	0.043	0.002**
ND4L	1.010	0.084	0.682	0.049	0.020*
ND5	1.013	0.096	0.812	0.099	0.196
ND6	1.012	0.096	0.883	0.084	0.350
CYTB	1.014	0.098	0.682	0.043	0.035*
			Plestiodon capito (8°C)		
COI	1.007	0.072	0.536	0.036	0.003**
COII	1.002	0.041	0.804	0.044	0.022*
COIII	1.002	0.038	0.632	0.025	0.001**
ATP8	1.084	0.114	0.937	0.041	0.540
ATP6	1.008	0.073	0.990	0.158	0.925
ND1	1.004	0.055	0.732	0.084	0.040*
ND2	1.012	0.086	1.164	0.066	0.212
ND3	1.011	0.085	1.151	0.182	0.537
ND4	1.006	0.063	0.343	0.014	0.001**
ND4L	1.004	0.051	0.861	0.092	0.236
ND5	1.007	0.067	0.752	0.020	0.027*
ND6	1.027	0.076	0.762	0.048	0.019*
CYTB	1.026	0.065	1.125	0.131	0.440
			Scincella modesta (8°C)		
COI	1.012	0.087	1.144	0.074	0.293
COII	1.005	0.058	1.173	0.064	0.099
COIII	1.004	0.050	1.475	0.033	0.000**
ATP8	1.013	0.059	1.328	0.083	0.046*
ATP6	1.016	0.110	0.844	0.043	0.221
ND1	1.020	0.117	0.655	0.041	0.046*
ND2	1.026	0.134	0.764	0.046	0.143
ND3	1.013	0.097	1.570	0.098	0.005**
ND4	1.020	0.116	0.906	0.095	0.479
ND4L	1.022	0.121	2.294	0.086	0.000**
ND5	1.000	0.017	1.291	0.079	0.032*

ND6	1.014	0.092	1.563	0.056	0.000**
CYTB	1.006	0.074	1.178	0.086	0.530
Scincella reevesii (8°C)					
COI	1.005	0.058	0.745	0.040	0.013*
COII	1.013	0.093	0.798	0.048	0.101
COIII	1.008	0.074	0.825	0.064	0.113
ATP8	1.047	0.062	0.396	0.040	0.000**
ATP6	1.007	0.071	0.984	0.102	0.511
ND1	1.010	0.082	1.105	0.107	0.512
ND2	1.002	0.039	1.194	0.094	0.132
ND3	1.015	0.100	0.856	0.101	0.279
ND4	1.018	0.115	0.976	0.074	0.772
ND4L	1.018	0.130	0.731	0.045	0.146
ND5	1.007	0.068	1.204	0.071	0.092
ND6	1.003	0.188	0.954	0.029	0.634
CYTB	1.004	0.052	0.918	0.051	0.281
Scincella modesta (4°C)					
COI	1.012	0.094	0.328	0.068	0.001**
COII	1.013	0.093	0.434	0.042	0.004**
COIII	1.013	0.096	0.269	0.031	0.003**
ATP8	1.033	0.151	0.385	0.042	0.019*
ATP6	1.034	0.153	0.478	0.061	0.029*
ND1	1.005	0.058	0.914	0.023	0.224
ND2	1.002	0.033	0.958	0.062	0.564
ND3	1.013	0.097	0.414	0.038	0.005**
ND4	1.027	0.143	0.530	0.098	0.033*
ND4L	1.024	0.128	0.840	0.099	0.300
ND5	1.023	0.126	1.777	0.168	0.013*
ND6	1.010	0.081	1.390	0.143	0.071
CYTB	1.024	0.122	0.449	0.033	0.015*

Table S1. Relative expression levels and P-values for 13 PCGs in four skinks.
