# Supplementary material for: Comparison of Mitochondrial Genome Expression Differences among Four Skink Species Distributed at Different Latitudes under Low-Temperature Stress
Source: Int J Mol Sci. 2024 Oct 2;25(19):10637. doi: 10.3390/ijms251910637 (PMC11605214; doi:10.3390/ijms251910637)
Supplement: Supplementary file 1 [file ijms-25-10637-s001.zip › Table S2.pdf]

Table S2. Modified universal primer details for sequencing the mitochondrial genomes of in this study [101]. Note: W = A/T, R = A/G, K = G/T, Y = C/T, S = G/C, M = A/C, B = C/G/T, D = A/G/T, H = A/C/T, N = A/C/G/T.

Primer Name	Nucleotide Sequence (5′ to 3′)
xiyi-12S-J505	ACAAACTAGGATTAGATACCC
xiyi-12S-N1225	CANBTTTCCCTTGCGGTACT
16SL	AACCCYYGTACCTYTTGCATCATG
16SH	TCCACAGGGTCTTYTCGTC
ND1-ND2L	CGATTTCGCTATGACCAACT
ND1-ND2H	ATTGATGAGWAKGCTATRATTTTTCG
ND2-CO1L	GCCCCMYTMCACTTCTGA
ND2-CO1H	GTAHAGGGTGCCRATRTCTTT
SX-ND5-ND6-J	GARCARGACCTYCGACTAATRGG
SX-ND5-ND6-N	ATATTAGTAGTGTTTGTSTATAC
CO1-CO2L	TACTCAGACTACCCAGAYGC
CO1-CO2H	CCRCARATTTCTGAGCATTG
ND4-CUNL	CCMAAAGCCCAYGTAGAAGC
ND4-CUNH	CTTHTACTTGGADTTGCACC
SX-ND5-GLU-J	YTYATTAACGCCTGAGCCTT
SX-ND5-GLU-N	ATAACAACGAYGGTTTTTC
CYTB-ProL	TGAGGACAAATATCMTTCTGAGG
CYTB-ProH	TTAAAATKCTAGTTTTGG
Thr-CRL	YAAAGCMTTGRTCTTGTAA
Thr-CRH	CTCGAKTTTWGGGGTTTGRCGA
CR-12SL	TCGYCAAACCCCWAAAMCGAG
CR-12SH	TRTAACCGCGGTKGCTGGCAC
