# Supplementary material for: Comparison of Mitochondrial Genome Expression Differences among Four Skink Species Distributed at Different Latitudes under Low-Temperature Stress
Source: Int J Mol Sci. 2024 Oct 2;25(19):10637. doi: 10.3390/ijms251910637 (PMC11605214; doi:10.3390/ijms251910637)
Supplement: Supplementary file 1 [file ijms-25-10637-s001.zip › Table S3.pdf]

Table S3. *RT*-qPCR Primer of the 13 mitochondrial PCGs and *β-actin* in this study. Note: "DLLW" means *P. elegans*, "DLHW" means *P. capito*, "DLNHX" means *S. reevesii* and "NBHX" means *S. modesta*.

Gene Name	Forward Primers (5' - 3')	Reverse Primers (5' - 3')
DLLW-*COI*	ACTTCCTTGGTCTGTCTGGTAT	GTCATTCTAGGTTGGTGCTTGT
DLLW-*COII*	TCCAACACAAGACCTACAACAG	GGACGGCTCAAGAGTGAAGTA
DLLW-*COIII*	GCCTTCTACCACTCCAGCTTA	TGTTACGGTCACGCCAGAG
DLLW-*ATP8*	CCTTGGTTCCTAATCCTCCTAC	GGTTGTGTGGTGGGTTGAC
DLLW-*ATP6*	ACCTCTTACCAGAAGGCACAC	AGTTAGGCTCGCTGTTGTAGT
DLLW-*ND1*	CCGTAGCCTTCCTAACACTCT	CCATCTGCGATTGGTTGTAGTA
DLLW-*ND2*	CAGCCGCAACAACAATAGC	GGTGTGATTGGTAGGAGAAGG
DLLW-*ND3*	CACCACTACCATCACCATCCTA	GGTCAAAGCCACACTCGTATG
DLLW-*ND4*	ACACAGCCGAACTCTATTACTC	AGGTTGATTGTTGGTGGAAGG
DLLW-*ND4L*	CCTTGTGTCTGCCTTACTCTG	CTATGGTGGTGGATGGTAGTTG
DLLW-*ND5*	CACATCTCAACGCACGCATT	ACAGGCAGGACGAGGTGAT
DLLW-*ND6*	TGTAGCGTCTAATCCATCTCCT	GCAAACACCACTAGCATCCC
DLLW-*Cytb*	TTCTTATTCGCATACGCCATCC	TGAGATTGGACGGAAGGTGTT
DLLW-*β-actin*	CCTTCAACACTCCAGCCA	CATACAGGGATAGCACAGCC
DLHW-*COI*	AATGAGACGCCGCAATACTATG	TACGATGTCCAGTGACGAGTT
DLHW-*COII*	CCAACACAGGACCTACAACAG	ATGAGTGGAGCACGTCTTCT
DLHW-*COIII*	GCAATCTCCGACAGCGTAT	CGTAATAGGCAGACAATCAAGA
DLHW-*ATP8*	CTGGTTTCTAATCCTCCTGCTA	GGGTTGTGTGGTGGGTTAG
DLHW-*ATP6*	GCACTTGGTGTCCGACTAAC	ATGGCAACAGCAATCTCTAGG
DLHW-*ND1*	ATGGCTCCTACTATGCTCTTGA	ATTCTGATTCGCCTTCGGTAAG
DLHW-*ND2*	CCTCCGCCACTGTGCTATT	TGCCTTGTAGGACTTCTGGTAA
DLHW-*ND3*	ACCCTATGAGTGTGGCTTCG	AGGATGATGATGGTGCTTGTTC
DLHW-*ND4*	GCCTGCCTACTAGCCTTCTTA	TGATGCGAATAATGCCGTATCC
DLHW-*ND4L*	CACACCTAGTCTCTGCCTTACT	TGGTTGAGGATGGAAGTTGGA
DLHW-*ND5*	CACTACTTCACTCAAGCACCAT	CGATACAGAGGCAGATTGTCAG
DLHW-*ND6*	CCAACCAGACACCAATAAGC	GTTCTTGGTTCGTGCTGATT
DLHW-*Cytb*	TACTACTCTTCCTCCTCCTCCT	GGATGGCGTATGCGAATAGG
DLHW-*β-actin*	ACCTTCAACACGCCAGCTA	ACACCATCACCAGAGTCCAT
DLNHX-*COI*	ACACCCGAGCATACTTTACCT	AGTCCGCCTACTGTGAATAGAA
DLNHX-*COII*	ACACAAGAACTACAACCAGGAA	AGCGTCTGTCTTAATACCAAGG
DLNHX-*COIII*	GACCACCAAGCGGAATCAAC	TAGGAGGACTGTAAGCAGAAGG
DLNHX-*ATP8*	CCTCCCCTTGGTTTCTTAT	GCGGTGTGATGTGCTATGA
DLNHX-*ATP6*	CCGCTGGTCATCTGTTAATTCA	TAGGCTTGGATCATTGCTACTG
DLNHX-*ND1*	TACCACTGACTACTCCTATGCT	CGTTGAAGCCAGATACAAGTTC
DLNHX-*ND2*	GAGCCACTGGAACCTGAGATA	GGTGAGCCTTGTAGGACTTCT
DLNHX-*ND3*	ACAGACAACACCAGACACAGA	CAGGGTTTGGTTGGTTTATTGC
DLNHX-*ND4*	TCTGACTAGCCTGCCTCCTA	GATTGCCGCTAAGACCATAGAC
DLNHX-*ND4L*	GCATCGCACGCATCTTGT	ATTGGAAGCAGGGTTGTAGTTG
DLNHX-*ND5*	GCTCTTACCGCCGCCTATA	GCCACAATGCTTCCTAATGCTA

DLNHX-*ND6*	GGTTGCTGTTGCGTCTAATCC	AGAACTGCCAAGTCACACCAA
DLNHX-*Cytb*	CCTGCTTGGAATCTGCCTAATC	GCGTCCGATGTGAAGGTAGA
DLNHX-*β-actin*	AGACCACCTTCACTCCATCAT	GTGTTGGCATACAGGTCCTTG
NBHX-*COI*	GAGCAGTGTTCGCAATTATGG	CTGAGTATCGTCGTGGTATTCC
NBHX-*COII*	ACACTTCCATGACCATGCTATC	CTCGACTTCTTGTGCGTCTATA
NBHX-*COIII*	GGCACATACCAAGGACATCATA	TCAGTAGCCGCCTAGTTCAG
NBHX-*ATP8*	GACACCCTTAGATAGCCCTACA	AAGTTCATGGTCAGTCTCATGG
NBHX-*ATP6*	CCAACCAACTCTATCTCTAGGC	GTAGAACGAATACGGCTGTAGA
NBHX-*ND1*	ACCTGACTCCTATTCTCCTCTT	CGGCTGCGTATTCTACATTGA
NBHX-*ND2*	TTCACTGACTACTTGCCTGAGT	TAGTGGTGGCTTCTGTTGCT
NBHX-*ND3*	GAGCAGTGTTCGCAATTATGG	CTGAGTATCGTCGTGGTATTCC
NBHX-*ND4*	ATGACTTCCGCACCACTAATG	CACAAGGTCGGTTGCTGAG
NBHX-*ND4L*	CCATACTACCCGTGATTCTCCT	CCGTGAGTTCGAGCAGTTG
NBHX-*ND5*	AACATTGCCAATCACCACCAG	AGGCTATATGCGGCGGTTAG
NBHX-*ND6*	CAACACAACCAACACCAATTCC	TTGCGTATTCTGTGGCGTTAG
NBHX-*Cytb*	ATCGGACGAGGACTATACTACG	GGTACGGCTGATAGGAGGTT
NBHX-*β-actin*	CCTTCAACACTCCAGCCATG	TACGACCAGAGGCATACAGG
